# Living with type 1 diabetes mellitus: experiences of Colombian teenagers and parents

**DOI:** 10.1590/1980-220X-REEUSP-2024-0340en

**Published:** 2026-03-02

**Authors:** Cristina Bohórquez Moreno, Alejandra Fuentes-Ramirez, Gloria Carvajal Carrascal, Diana Obando Posada, Paola Duran Ventura, Robin Whittemore

**Affiliations:** 1 Universidad de La Sabana, Facultad de Ciencias de la Vida y el Bienestar, Chía, Colombia.; 2 Universidad de La Sabana, Facultad de Ciencias del Comportamiento, Chía, Colombia.; 3 Fundación Cardioinfantil-IC, Bogotá, Colombia.; 4 Endociencia, Bogotá, Colombia.; 5 Yale University, New Haven, United States.

**Keywords:** Diabetes Mellitus, Type 1, Adolescent, Parents, Family Relations, Life Change Events, Nursing, Diabetes Mellitus Tipo 1, Adolescente, Pais, Relações Familiares, Acontecimientos que Cambian la Vida, Enfermagem

## Abstract

**Objective:**

To explore the experiences of Colombian adolescents and their parents after a type 1 diabetes mellitus (T1D) diagnosis.

**Method:**

A descriptive qualitative study using content analysis was conducted in Bogota, Colombia. Between May and June 2024, a total of 36 participants were enrolled, distributed into two focus groups with 16 adolescents and three focus groups with 23 parents. Semi-structured interview transcripts were analyzed systematically to identify common themes across all participants. The study adhered to ethical principles in research.

**Results:**

Six themes were identified: (1) experience of an initial emotional impact, (2) changes in family dynamics, (3) the need to learn diabetes management, (4) reliance on external support, (5) the importance of empathetic and knowledgeable healthcare professionals, and (6) ongoing challenges in managing the condition.

**Conclusion:**

The findings highlight that living with T1D requires emotional, behavioral, and social adaptation. Comprehensive, family-centered interventions that foster resilience, self-management, and emotional support are essential to improving outcomes for both adolescents and their families.

## RESUMO

**Objetivo:**

Explorar as experiências de adolescentes colombianos e de seus pais após o diagnóstico de diabetes mellitus tipo 1 (DM1).

**Método:**

Foi realizado um estudo qualitativo descritivo com análise de conteúdo em Bogotá, Colômbia. Entre maio e junho de 2024, participaram 36 pessoas distribuídas em dois grupos focais com 16 adolescentes e três grupos focais com 23 pais. As transcrições das entrevistas semiestruturadas foram analisadas sistematicamente para identificar temas comuns entre todos os participantes. O estudo seguiu os princípios éticos em pesquisa.

**Resultados:**

Seis temas foram identificados: (1) vivência do impacto emocional inicial, (2) mudanças na dinâmica familiar, (3) necessidade de aprender o manejo do diabetes, (4) dependência de apoio externo, (5) importância de profissionais de saúde empáticos e bem informados e (6) desafios contínuos no manejo da condição.

**Conclusão:**

Os achados destacam que viver com DM1 requer adaptação emocional, comportamental e social. Intervenções abrangentes e centradas na família que promovam resiliência, autocuidado e apoio emocional são essenciais para melhorar os resultados tanto para adolescentes quanto para suas famílias.

## INTRODUCTION

Type 1 Diabetes (T1D) is a chronic health condition in which the pancreas is unable to produce insulin. According to the International Diabetes Federation (IDF) for the year 2022, approximately 8.75 million people were living with this condition, of whom 1.52 million were under 20 years old^(1,2)^. In Colombia, the incidence of T1D is 6 to 7 per 100,000 children, and specifically in Bogotá, approximately 400 children and/or adolescents are diagnosed each year^(3,4)^. This condition imposes a high cost on the healthcare system due to long-term complications^(5)^.

A T1D diagnosis during adolescence presents unique challenges due to the developmental stage in the life cycle characterized by physiological and psychosocial changes, which are intensified by the coexistence of chronic illness. Adolescents may experience symptoms of stress, depression, and anxiety, directly affecting glycemic control and thereby negatively impacting their quality of life^(6,7)^. Managing diabetes requires significant adjustments for both the adolescent and their family, including activities such as blood glucose monitoring, insulin administration, and adjustments to diet and physical activity. These changes can disrupt the dynamic between adolescents and their parents, who are primarily responsible for their children’s daily care, consequently contributing to stress and anxiety due to a lack of knowledge about the condition, different priorities of adolescents and parents, and the changes it imposes on family dynamics^(6,8,9)^. This process generates varied demands for both adolescents and parents, such as educational support, emotional support, and healthcare-related needs.

Managing T1D is complex and continuous education is required for both adolescents and their parents on glucose control, carbohydrate intake, and consistent insulin dosage adjustments based on the adolescent’s diet and physical activity. During adolescence, hormonal changes can significantly alter blood glucose levels, adding another challenge to managing the disease. Furthermore, developing autonomy during this life stage can hinder adherence to treatment guidelines, especially without adequate family support. Therefore, it is essential for family members to also receive training to provide necessary support^(10,11)^.

Adequate family support for adolescents can lead to better quality of life, greater emotional well-being, improved self-care, and adherence to treatment. However, it is not easy for families to assume responsibility for T1D care, as it requires significant lifestyle adjustments and involves an intensive care regimen that families are often unfamiliar with^(8)^. A comprehensive approach by an interdisciplinary team that involves adolescents and their parents can facilitate adaptation to T1D, prevent complications, build a new normal within the family, and create a healthy home environment^(12,13)^.

In the literature reviewed from the past 10 years in Colombia, there is limited research aimed at understanding the experience of adolescents and their parents following a T1D diagnosis, particularly from a qualitative perspective. Little is known about how families manage T1D daily and unmet needs that could inform culturally relevant interventions. While considerable research has been conducted globally on the experience of adolescents and parents living with T1D, such findings may not fully capture the Colombian context. Differing health care systems and cultural values, beliefs, and traditions can significantly shape how a complex chronic illness like T1D is experienced and managed. Therefore, research grounded in local realities is critical to provide culturally relevant insights and support strategies^(9)^. Understanding the factors influencing Colombian families on diabetes self-management can inform culturally relevant interventions and future research. The purpose of this study was to explore the experiences of Colombian adolescents and their parents after a type 1 diabetes diagnosis.

## METHOD

### Study Design

This is a descriptive qualitative study guided by content analysis^(14)^. Data were collected through focus group interviews. The study followed the Consolidated Criteria for Reporting Qualitative Research (COREQ) checklist to ensure transparency and rigor in the reporting process^(15)^. The aim of the study was to explore the experiences of adolescents diagnosed with type 1 diabetes mellitus and their parents, particularly in relation to the challenges encountered after diagnosis and during disease management.

### Place

The study was carried out in Bogotá, Colombia, between May and June 2024. The face-to-face focus groups took place at the facilities of the Colombian Diabetes Association. Virtual sessions were conducted via the Microsoft Teams^®^ platform, allowing flexibility and accessibility for participants located in different areas of the country.

### Population and Selection Criteria

The study included 39 participants: 16 adolescents aged 10 to 17 years with a confirmed diagnosis of T1D, and 23 parents of adolescents with T1D. The inclusion criteria required the ability to read and write in Spanish and a willingness to participate in a focus group, either in person or virtually. A convenience sampling strategy was employed, and participants were intentionally selected to form focus groups with homogeneity in age, sex, and geographical location, thereby facilitating the expression of shared experiences.

Despite this intentional grouping, the overall sample was heterogeneous, encompassing diverse socioeconomic and educational backgrounds. For context, the study focused on adolescents from diverse family backgrounds and social environments, including different family compositions, parental education levels, and residential settings in Bogotá and other cities. This diversity provides a comprehensive perspective on the experiences and contexts relevant to the research topic.

Fifty families receiving care at two diabetes foundations were invited to participate by telephone. Of these, 23 agreed to take part in the study. The main reason for non-participation was lack of time. The invitation and explanation of the study objectives were conducted by the principal investigator, who ensured confidentiality and voluntary participation.

### Data Collection

To encourage open expression of experiences, adolescents and parents were interviewed separately. The focus group sessions followed established guidelines and included the following steps: a) selecting experienced moderators, b) designing a semi-structured question guide, and c) organizing five focus groups^(16)^. The semi-structured question guide was developed by a multidisciplinary team with experience in nursing, public health, clinical psychology, and Colombian healthcare services, with the aim of exploring the experiences and needs arising with a T1D diagnosis. The focus group sessions lasted between 40 and 90 minutes. All sessions were audio-recorded and transcribed verbatim, with participants’ names excluded to ensure confidentiality, and each participant assigned a code number. Field notes were taken on methodological and logistical aspects, and participants were asked to complete a brief sociodemographic questionnaire including questions about participants age, sex, socioeconomic status, family structure, among others^(17)^.

### Data Analysis and Treatment

For qualitative analysis, a content analysis approach was used, which involved systematically coding and categorizing the data^(14)^. To ensure methodological rigor, the principal investigator coded all interviews and held frequent meetings with the multidisciplinary team to review the coding process and resolve discrepancies. The team then organized the codes into categories and subcategories to identify key themes, utilizing Microsoft Word and Microsoft Excel for data management. A systematic approach was used to ensure consistency in coding and interpretation across the dataset.

Credibility was ensured through the use of in vivo codes, collaborative analysis, and validation of results with participants using the “member-checking” technique. Additionally, theoretical saturation was reached, ensuring that no new themes or insights emerged from the data, reinforcing the depth and comprehensiveness of the analysis. In this approach, consolidated findings were returned to participants for accuracy checking. Furthermore, methodological and theoretical triangulation strategies were applied to enhance the transferability of the results^(18)^. This process yielded expressions such as the following:

Participant 1: *Excellent, definitely. You managed to summarize everything beautifully, and indeed, as I have always said and always suggested to the association, to empower these children and make them productive in the future, we need to strengthen the backbone of this whole situation, which is the mothers. Thank you very much; it was really very touching*.

Participant 2: *A perfect synthesis of everything that happens in the process following diagnosis, and then realizing they have to live with a chronic condition for life. I think it is very well done*.

### Ethical Aspects

The study adhered to the ethical standards outlined in Resolution 008430 and received ethical approval under record number 003 on February 19, 2024, from the Ethics Committee of the School of Nursing and Rehabilitation, as well as Minute No. 202 on May 22, 2024, from the Faculty of Behavioral Sciences at Universidad de La Sabana. Written informed consent was obtained from the parents, and written informed assent from the adolescents. An alphanumeric coding system was employed to ensure participant anonymity.

## RESULTS

Sixteen adolescents with T1D participated in the study, with a mean age of 13.6 years (SD = 2.4). Among them, 68.8% (n = 11) were female. Most adolescents were enrolled in the tenth grade, representing 31.3% (n = 5) of the sample; regarding socioeconomic status, 43.8% (n = 7) were of a low socioeconomic stratum according to the Colombian classification. Additionally, half of the participants (50.0%, n = 8) reported living with only one parent. The majority (81.3%, n = 13) resided in Bogotá (Table 1).

**Table 1:** Sociodemographic characteristics of adolescents – Colombia, 2024.

Variable	Categories	N (16)	%
Categorical Variables			
Sex	Male	5	31.3
	Female	11	68.8
Academic Grade	Fifth	2	12.5
	Sixth	2	12.5
	Seventh	1	6.3
	Eighth	1	6.3
	Ninth	3	18.8
	Tenth	5	31.3
	Eleventh	2	12.5
Socioeconomic Status	Low	7	43.8
	Lower-middle	5	31.3
	Upper-middle	3	18.8
	Upper	1	6.3
Family Structure	Mother and father	6	37.5
	Only mother or father	8	50.0
	Other people	2	12.6
Place of Residence	Bogotá	13	81.3
	Other cities	3	18.9
Continuous Variables			
	Mean (SD)	Min–Max	
Age (years)	13.6 (2.4)	10–18	

Source: Own elaboration.

In addition, twenty-three parents of adolescents with T1D also participated in the study, of whom 82.6% (n = 19) were female. In terms of educational attainment, 30.4% (n = 7) had completed technical education, followed by 21.7% (n = 5) with secondary education, and 21.7% (n = 5) with university education. Regarding socioeconomic status, most parents were of a low socioeconomic stratum (34.8%, n = 8), according to the Colombian classification. Marital status was distributed as follows: 34.8% were married, 26.1% were single, and 13.0% were either divorced, in a common-law union, or separated. In terms of family composition, 65.2% reported living with both parents and children, 21.7% with one parent and children, and 13.0% with grandparents or other household members. The majority (73.9%) resided in Bogotá, while 26.1% lived in other cities (Table 2).

**Table 2:** Sociodemographic characteristics of parents – Colombia, 2024.

Variable	Categories	N (23)	%
Categorical variables			
Sex	Male	4	17.4
	Female	19	82.6
Educational level	Incomplete Secondary	4	17.4
	Complete Secondary	5	21.7
	Technical Training	7	30.4
	University	5	21.7
	Postgraduate	2	8.7
Socioeconomic status	Lower-low	1	4.3
	Low	8	34.8
	Lower-middle	7	30.4
	Upper-middle	3	13.0
	Upper	3	13.0
	High	1	4.3
Marital status	Single	6	26.1
	Married	8	34.8
	Divorced	3	13.0
	Common-Law Union	3	13.0
	Separated	3	13.0
Family structure	Mother, father, and Children	15	65.2
	Only mother or father and children	5	21.7
	Mother, father, children, and grandparents	1	4.3
	Other people	2	8.7
Occupation	Student	2	8.7
	Employed	9	39.1
	Unemployed	7	30.4
	Homemaker	3	13.0
	Self-employed	2	8.7
Place of residence	Bogotá	17	73.9
	Other cities	6	26.1
Continuous variables			
	Mean (SD)	Min–Max	
Age (years)	45.3 (9.5)	30–68	

Source: Own elaboration.

### Analysis: Themes

**Figure 1:**
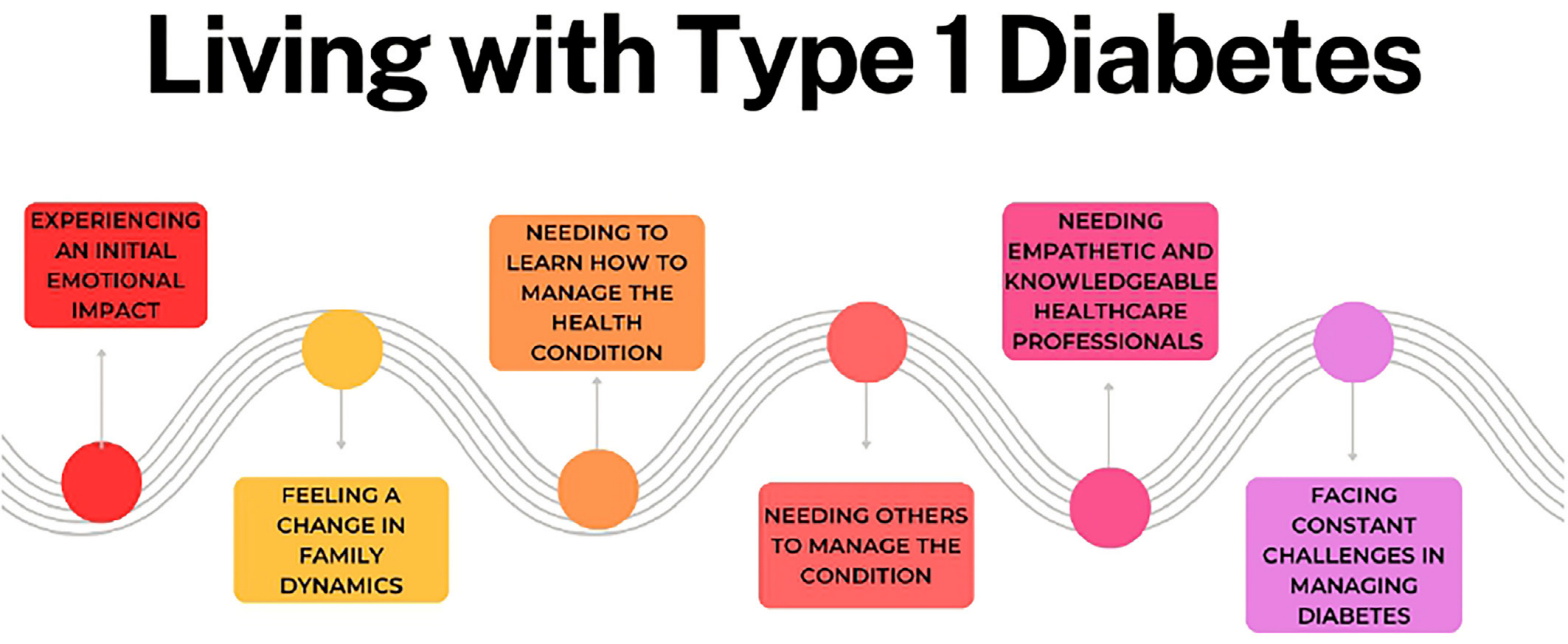
Living with T1D: adolescent and parent perspective.

Source: Own elaboration.

Participants reported that living with T1D was a complex process characterized by multiple challenges and necessities. This process, illustrated in Figure 1, begins with a significant emotional impact on both adolescents and their parents. It is followed by changes in family dynamics and the need to develop skills to manage the disease. These themes are interconnected, forming a conceptual framework that reflects the participants’ lived experiences. In the data analysis, common themes, rather than differences, of both adolescents and parents were identified. This representation highlights key stages and challenges, allowing for a holistic understanding of participants’ experiences.

### Theme 1: Experiencing an Initial Emotional Impact

Adolescents and parents in this study reported that the diagnosis of T1D had a major impact on their lives.

Parents of teenagers often experienced complex emotions at the time of diagnosis, including fear and confusion due to their limited understanding of how to manage this complex health condition. They frequently expressed constant worry about potential complications and also felt guilty for being unable to prevent the challenges their children faced as a result of the diagnosis. These feelings of fear, doubt, and guilt were intensified by the lack of knowledge and the traumatic situations experienced by both the parents and their children in hospitals or during the initial diagnosis and treatment.

Below are some examples of parent codes:

Dad 1: *J debuted at 10 years old, mmm, it was always an impact for us because he was in the ICU. So, he was in the ICU. So, it got a little complicated, so it was a bit complex from that side*.

Mom 4: *Then I broke into tears. I really broke into tears. I said, ‘My God, but why?’ I said, ‘I did everything.’ I blamed myself*.

Mom 7: *It’s difficult—with bullying, without bullying, with love, with sports, without sports, in any circumstance, this is very difficult. With money, without money, I mean, it’s difficult from any point of view*.

Teenagers often experienced feelings of confusion and struggled to adapt to life with T1D. Initially, they reported feelings of resistance and denial toward the diagnosis. Coping with lifestyle changes was particularly challenging, especially due to dietary restrictions, the regular use of insulin and glucometers, and the need for continuous self-monitoring. Additionally, they felt a heightened sense of responsibility and could become overwhelmed by the demands of managing their condition. This situation was further complicated by interactions with their friends and peers, as some teenagers experienced bullying at school. These negative social experiences added an emotional burden, making it even more difficult for them to adapt to their new reality.

Adolescent 2: *Well, for me, it was a bit hard to get used to because sometimes I want to eat sweets or something, and I can’t. I wouldn’t inject my insulin or check my glucose levels, but I learned with my mom because I saw her getting tired of fighting for me*.

Adolescent 4: *I’m 15 years old. Well, for me, it was hard, but now at school, some classmates help me. Many of them also remind me to take everything I need, so they help me a little*.

Adolescent 5: *Well, sometimes you feel pressure because if you make a mistake, you feel bad since a mistake can cause hypoglycemia, so it’s difficult*.

Adolescent 8: *It was a little hard, but I thought I didn’t have a problem. However, it’s always difficult with classmates. Sometimes they want to bully you, and those words hurt*.

### Theme 2: Feeling a Change in Family Dynamics

The diagnosis of T1D contributed to significant changes in family dynamics, particularly related to lifestyle adjustments, with special emphasis on diet and sleep and rest habits.

Parents reported experiencing shifts in their daily routines, with the constant concern for proper disease management becoming a 24-hour responsibility. This new dynamic often affected their work, rest, and social life. Additionally, family conflicts could arise due to the challenges associated with adolescence, with parents perceiving themselves as overly strict or overprotective, which created tension in their relationship with their child. Despite these difficulties, parents expressed a strong commitment to caring for and protecting their children. They adapted to these changes and actively sought solutions to improve their family’s well-being.

Below are some examples of parent codes:

Dad 2: *It has caused some conflict because I end up being the strict dad, the angry dad. ‘Why haven’t you taken your insulin? What’s going on? Do you want to be in the hospital?*

Mom 4: *At home, if there are forbidden foods, it’s because they are not in the house—it’s that simple. The rest of the food in the house, we can all eat*.

Mom 5: *Usually, on those days, because of physical activity, at night when they are resting, their levels tend to drop, and drop, and drop, and drop. So, it’s 1, 2, 3, 4 times waking up, and you know that interrupted sleep is exhausting*.

Teenagers report that after being diagnosed with T1D, their parents emphasize that they have to take responsibility for their own care. For example, they must monitor their glycemic levels regularly, often interrupting their daily activities in the social and school settings. Thus, adolescents constantly adapt to new challenges, seeking balance in their daily lives.

Adolescent 3: *At school, my parents are always asking me for my glucose readings, telling me to send them my glucose readings, and sometimes it gets annoying*.

Adolescent 4: *I think it’s also kind of annoying for me because they’re always there, controlling, watching, checking if I took my glucose reading, if I injected my insulin*.

### Theme 3: Needing to Learn to Manage Health Condition

Adolescents and parents perceived T1D management as a complex process that required comprehensive education from healthcare professionals. They felt that education was essential to help them adapt lifestyle changes associated with T1D. Both adolescents and parents expressed the need to learn how to correctly dose insulin units, count carbohydrates, and manage hyperglycemic and hypoglycemic crises effectively.

Parents, in particular, reported that they required comprehensive and continuous education starting from the time of diagnosis. Many reported a lack of clear and detailed guidance on managing their children’s condition, particularly in critical areas such as carbohydrate counting, dietary management, insulin administration, and the use of technologies like insulin pumps and glucometers. Additionally, parents had to develop strategies for positive parenting, fostering autonomy and avoiding fear-based approaches. To fill these gaps, parents often sought information through social media and support groups to counter the stigma and misinformation in their environment. Parents emphasized the importance of structured and accessible educational programs addressing both the medical and psychosocial aspects of diabetes management in adolescents, as they felt these programs would have helped them navigate the challenges of T1D with more confidence and competence.

Below are some examples of excerpts from teens and parents:

Mom 4: *There isn’t much training on carbohydrate counting, uh, how to manage that*.

Mom 7: *When the child is diagnosed, no one tells you what to do. They just tell you, ‘Check their glucose levels. If it’s within this range, then that’s it,’ but no one really educates you as a parent*.

Mom 6: *So, well, I don’t know, I feel that the ideal thing would be for this not to become just another routine but for the education given to parents of children with this condition to be truly engaging, in a playful way, so that they also get involved and learn*.

Adolescents expressed the need for continuous and comprehensive guidance regarding their condition, even years after their initial diagnosis, emphasizing it should be accessible, friendly, and detailed, including information about treatment, practical advice, and solutions for daily management. They highlighted the importance of appropriate nutritional education, particularly with a focus on carbohydrate counting. Additionally, they underscored the essential requirement of psychoeducation within their social environment, aimed at raising awareness about the risks and management of situations such us hypoglycemia and hyperglycemia to ensure a safe and supportive environment.

Adolescent 3: *“Sometimes we need guidance because, even though we’ve had diabetes for many years, we still don’t know everything it requires.”*

Adolescent 4: *A more family-friendly introduction, because in the group, sometimes the moms who have newly diagnosed children just get the diagnosis, but they don’t get a complete introduction to what diabetes really is, something comfortable, with tips, solutions, and all that*.

Adolescent 5: *I think what is really missing is education about food, because sometimes they restrict everything, and you basically can’t eat anything at all. I believe they should focus more on teaching how to count carbohydrates to avoid completely restricting food*.

### Theme 4: Needing Social Support to Cope with Living with T1D

Adolescents and parents reported that social support was important for the adaptation to T1D. Parents of adolescents expressed the need for emotional support to cope with the constant stress and anxiety associated with caring for their children. They emphasized the relevance of connecting with other parents in similar situations to share experiences and gain reassurance. They also required support for the daily management of diabetes, including tasks such as nighttime monitoring, medication administration, and carbohydrate counting. Parents emphasized that family and social support were critical for maintaining their own mental well-being. Many parents also reported finding strength in their spiritual beliefs, which provided them with comfort and guidance throughout the adaptation process. Below are some examples:

Mom 5: *Especially because my dad almost passed away six months ago and he was virtually both a father and grandfather to my daughter. So, my dad also played a big role; he was a fundamental support for us because he was always making sure about the legal procedures*.

Mom 6: *We met more moms, and that guidance was vital, truly vital, because now my daughter has some friends with the same condition. So, on weekends, I would invite them over, talk to their moms, and we would get together on the weekend*.

Mom 7: *You surrender to God, yes, I tell you, we surrender ourselves, as they say, to God, asking Him to help us, to give me strength, to give me peace so I could make wise decisions when I needed to make them*.

Adolescents reported experiencing social pressure that led to emotional distress, fostering a demand for social support that makes them feel comfortable sharing their experiences with peers facing similar situations. They strived to be accepted, feel understood, and maintain a sense of normalcy in their social environment. However, they noticed that it is often difficult for others to fully understand the complexity of living with T1D. While teenagers acknowledged that adapting to the diagnosis of diabetes was challenging, they emphasized the crucial role of their parents’ support in helping them adjust to the new reality of living with it.

Below are examples of codes for teenagers:

Adolescent 3: *At school, I never had friends because they always told me this was contagious, even though it’s never contagious, or they would call me the ‘weird girl*.

Adolescent 6: *More like putting yourself in our position as teenagers, because we want to live as normally as possible, and sometimes we get careless. We forget that we have a disease and that we have to be responsible—so, something like that*.

Adolescent 3: *Diabetes is not a disease; it is a condition that God sends for a life purpose, and I feel that accepting it was not that easy either*.

### Theme 5: Needing Empathetic and Prepared Health Professionals

Parents and adolescents reported predominantly negative experiences with the health professionals involved in their initial treatment.

Parents expressed that empathetic health professionals who are well-versed in managing T1D could have played a key role in helping them understand and adapt to the health challenges associated with the condition. They highlighted a lack of adequate professional support, noting that many health professionals did not provide clear and comprehensive guidance on managing the condition. They also observed that some professionals lacked the empathy and sensitivity needed to care for them and their children, describing certain interactions as “abrupt and unsympathetic”. As a result, many parents turned to private professionals or foundations to obtain the guidance and support that was often absent within the public healthcare system.

Here are some examples:

Mom 5: *Uh? I took her to the clinic, and the doctor who attended us was extremely rude. He told me straight to my face that my daughter could practically die, and it was a shock. I think all moms, when they are told, ‘Your daughter has this and she could die,’ we feel like it’s a huge blow*.

Dad 3: *When we had to have a blood test, the first one was done through the health insurance. The lab technician was also very rude, she yelled at the child because she saw him crying. I think in that aspect there should be a little more sensitivity with children*.

Mom 5: *I feel that, yes, what is truly essential is to find professionals who have ethics, who truly love their profession, and who get involved in the process of a disease that today is so common*.

Mom 4: *The psychologist we went to, only one appointment with the psychologist, and I didn’t like it. I didn’t like the treatment or the way we were treated… I said no, my baby is just a little baby… The nutritionist at the health insurance company, either…. I never went twice…. That woman was going to starve my daughter to death; basically, she couldn’t eat anything, just water*.

Adolescents also reported they required understanding, emotional support, and more empathetic communication from health professionals. They prefer professionals who provide ongoing guidance and support in managing the emotional and social challenges of living with diabetes.

Here is one example:

Adolescent 1: *I think there should be support to understand that, apart from that, let’s say, when we don’t know someone who has properly guided us on insulin application, how to have control, the diet, and carbohydrate counting*.

### Theme 6: Living with Constant Challenges in Diabetes Management

Managing T1D posed significant challenges for adolescents and their parents due to the constant fluctuations in blood sugar levels, insulin management, and carbohydrate counting required for dietary control.

Parents of adolescents with diabetes faced additional challenges in managing the condition across various social settings. They often relied heavily on monitoring practices such as continuous glucose monitoring, blood glucose testing, and others to keep their children’s blood glucose levels within the target range. Social events outside the home were particularly difficult, as parents found it challenging to estimate the carbohydrate content of foods they had not prepared, which led to anxiety and the need for contingency plans. Moreover, parental anxiety increased when adolescents participated in independent activities, such as social gatherings, as this required trusting their children’s ability to manage the condition effectively. Here are some examples:

Mom 4: *However, in her lunchbox, she carries insulin. In her lunchbox and her things, she carries the glucose meter, needles, we take all these precautions. If she needs to inject herself, she already knows how to do it*.

Mom 2: *Because there comes a time when adolescence arrives, trips with friend’s start happening. That was probably the most chaotic moment I have ever experienced. This dilemma was so overwhelming that I said, ‘No, take whatever you want.’ He is responsible for taking care of himself; he knows perfectly well how to manage himself*.

Adolescents faced numerous challenges in managing diabetes, including injecting insulin and testing their glucose levels at school, often in environments where they lacked adequate support. They also encountered daily difficulties related to food consumption, as most people are unfamiliar with carbohydrate counting. Adolescents reported having to bear the responsibility of balancing their desire to enjoy the foods they like with the need to maintain blood sugar control. This could lead to difficult decisions and feelings of isolation.

Adolescent 2: *Well, for me, it was a bit hard to get used to because sometimes I want to eat sweets or something, and I can’t. I wouldn’t take my insulin or check my glucose levels, but I learned with my mom because I saw her getting tired of fighting for me*.

Adolescent 5: *It’s very easy to make mistakes when counting carbohydrates, and even more so with what I’m telling you. If you could just serve a perfectly measured portion of rice, the meat, and everything exactly at 35 grams, it would be perfect*.

## DISCUSSION

In this research, six central themes were identified regarding the experiences and needs of Colombian adolescents and parents living with T1D. The first theme highlights that, at the time of diagnosis, both parents and adolescents faced a difficult situation due to a complex chronic illness, such as lack of knowledge about the disease and its management, which created physical and emotional challenges. These findings align with a study conducted in Brazil, which reported that when adolescents are diagnosed with diabetes, they undergo a complex process involving hospitalizations, medical tests, and the profound impact the diagnosis has on their lives^(18)^.

Similarly, in research conducted in Brazil, the diagnosis of T1D triggered feelings of worry, sadness, and anger among participants due to the unexpected nature of the situation and their limited knowledge about the disease, leading to moments of emotional distress^(19)^. Managing T1D poses significant challenges for families, as the emotional impact, lack of experience, and parental uncertainty in managing the condition can intensify the grief associated with accepting the diagnosis. This often interferes with daily life and may require specialized support^(20)^.

The second theme identified in this study focused on the changes experienced by adolescents and parents during the diagnostic process. These changes included adjustments to diet, physical activity, medication, and sleep habits. Previous research has shown that a T1D diagnosis significantly alters the lives of adolescents and their families. Adolescents must adopt new habits while often facing challenges in adhering to treatment. Dietary restrictions, constant glucose monitoring, and regular check-ups can result in difficulty with self-care responsibilities^(21)^.

The third theme emphasized by participants was the ongoing need for education on topics such as carbohydrate counting, dietary management, insulin administration, and the use of technologies such as insulin pumps and continuous glucose monitoring. Educating families on how to care for adolescents with a chronic condition not only alleviates the caregiving burden but also improves their quality of life^(19)^. However, education is not a one-time event; as children and adolescents grow, their developmental and medical requirements evolve, requiring continuous access to reliable educational resources. Research underscores the importance of ongoing diabetes education programs that provide updated information and support tailored to different life stages^(20)^. Providing clear and tailored information to parents and adolescents about managing these changes can help them better understand their situation, reduce emotional dysregulation, and promote adherence to treatment, ultimately improving metabolic control^(8,11,21)^.

The fourth theme identified the emotional support required by adolescents and parents. Both groups expressed the importance of receiving support from family members and others facing similar challenges to better adapt to life with diabetes. Similarly, a study conducted in China found that participants valued emotional support from family and peers with similar experiences, as it provided opportunities to discuss and learn strategies for managing diabetes. Addressing the concerns and demands of adolescents and their caregivers, and creating spaces for open discussion, is essential^(22,23,24)^. Social support has been recognized as a critical tool for enhancing the emotional well-being of adolescents with T1D. Strong family support positively influences self-efficacy and emotional well-being, mitigating the stress of living with the disease^(23)^. This need for support extends beyond the initial diagnosis to the entire management process. Professional organizations, local social support groups, and online networks play a pivotal role in connecting parents and adolescents with T1D, offering emotional and educational support. In addition to professional platforms such as the American Diabetes Association, community-based support groups provide culturally relevant guidance and a sense of belonging. Social media groups also facilitate experience-sharing, which promotes adaptation and improves treatment adherence^(25,26)^.

The fifth theme highlights the importance of competent and empathetic healthcare professionals in helping families adapt to T1D. This finding aligns with previous research, where adolescents reported insufficient support at the time of diagnosis and feelings of being overwhelmed by responsibility^(27)^. Specialized healthcare providers must be able to explain the interplay between glucose, diet, and insulin while providing specific nutritional recommendations^(10,25,28)^. In one study, adolescents with T1D described negative experiences, including depersonalized interactions and communication focused primarily on their parents. They also emphasized the need for more time to discuss their concerns with healthcare providers^(27)^.

Finally, the sixth theme addressed the challenges faced by parents and adolescents, including stress related to managing diet and blood glucose levels, particularly during social events where carbohydrate counting is difficult. Previous research noted that participants struggled to adhere to their diet in environments with sweets and sugary drinks, with these types of foods being especially tempting to adolescents^(28)^. Recently, advances in carbohydrate counting have enabled adolescents to eat a wider variety of foods^(27)^. However, as with adolescents without diabetes, this can lead to excessive calorie consumption and weight gain. It is therefore essential to understand the challenges adolescents and parents face regarding dietary restrictions and insulin injections, as these can impact adolescents’ self-image, adaptation, and daily activities. Providing effective coping strategies for managing T1D is crucial^(28)^.

The findings of this study provide valuable insights for healthcare professionals to design and implement interventions tailored to the specific exigencies of a South American population. A comprehensive, multidisciplinary approach is essential, with particular emphasis on supportive interventions at the time of diagnosis and ongoing education. Additionally, offering community-based and online resources for parents to receive emotional and social support may help both adolescents newly diagnosed with T1D and their families. Greater attention to routine screening for psychological distress, depressive symptoms, and anxiety in clinical care is also regarded as essential in established standards of care guidelines^(29)^. Addressing these demands through continuous education, psychosocial support, and specialized professional care will be crucial to meeting the holistic needs of this population in South American countries.

By focusing on Colombian adolescents and their families, this study contributes a culturally specific perspective that adds depth to the global understanding of T1D. Colombian families tend to be highly involved in the day-to-day management of chronic conditions, with both parents playing an active role in supporting adolescents. This underscores the necessity of educational interventions that include the entire family unit, rather than focusing solely on the adolescent. Furthermore, Colombian adolescents may benefit from routine psychosocial evaluation in clinical settings. While screening for depressive symptoms, diabetes-related distress, and anxiety is recommended in international standards of care, it is not consistently implemented in Colombia, and referral pathways to supportive services for affected adolescents remain limited. These findings highlight the relevance of strengthening the Colombian healthcare system by integrating family-centered education, psychosocial assessment, and access to appropriate mental health support into the routine care of adolescents with T1D.

Previous interventions have aimed to promote self-care behaviors and metabolic control in adolescents^(5,30)^, as well as family-focused strategies designed to improve the quality of life for both adolescents and their parents, enhance family communication, and foster problem-solving skills^(27,28,29)^. These findings underscore the need for further development and implementation of holistic approaches that integrate medical management, preventive care, education, and emotional support in healthcare systems worldwide. In some countries, nurses can become certified as diabetes educators, providing the comprehensive support adolescents with T1D and their families require, while in others, nutritionists play a key role in teaching skills such as carbohydrate counting. Exploring the availability and integration of these resources within the Colombian healthcare system could enhance the quality of care and outcomes for this population. Future research should be conducted on the implementation of comprehensive, multidisciplinary care in different healthcare systems, and evidence-based family-focused strategies should be modified in different cultural contexts.

Finally, it is important to consider the methodological limitations of this study when interpreting the results. The social dynamics of focus groups may have led to social desirability bias, where participants adjusted their responses to conform to group norms or the perceived expectations of the moderator. Additionally, the small sample size from a single geographical area limits generalizability; however, the findings are consistent with existing literature and can guide the development of culturally relevant interventions.

## CONCLUSION

The experience of living with T1D is shaped by significant emotional and behavioral adjustments for both adolescents and their parents. For adolescents, the diagnosis disrupts their sense of normalcy, leading to feelings of uncertainty, frustration, and resistance to treatment. They face challenges in maintaining social interactions, adhering to dietary restrictions, and integrating glucose monitoring into their daily lives. Parents, on the other hand, experience heightened anxiety, fear of complications, and an overwhelming sense of responsibility for their adolescent’s well-being. The constant need for vigilance, coupled with concerns about their child’s independence and future, often leads to emotional distress. The lack of comprehensive family-based education on T1D management further exacerbates these challenges, making daily care a source of tension within the family. Interventions that address both emotional adaptation and self-management skills are crucial in promoting a more positive experience of living with T1D, fostering resilience, and improving the quality of life for both adolescents and their parents.

## Data Availability

The entire dataset supporting the results of this study is available upon request to the corresponding author.
