# Supplementary material for: Living with type 1 diabetes mellitus: experiences of Colombian teenagers and parents
Source: Rev Esc Enferm USP. 2026 Mar 2;60:e20250340. doi: 10.1590/1980-220X-REEUSP-2024-0340en (PMC13004577; doi:10.1590/1980-220X-REEUSP-2024-0340en)
Supplement: Translation

# Translation: Vivir con diabetes mellitus tipo 1: experiencias de adolescentes y padres colombianos

**DOI:** 10.1590/1980-220X-REEUSP-2024-0340es

**Authors:** Cristina Bohórquez Moreno, Alejandra Fuentes-Ramirez, Gloria Carvajal Carrascal, Diana Obando Posada, Paola Duran Ventura, Robin Whittemore

**Affiliations:** 1 Universidad de La Sabana, Facultad de Ciencias de la Vida y el Bienestar, Chía, Colombia.; 2 Universidad de La Sabana, Facultad de Ciencias del Comportamiento, Chía, Colombia.; 3 Fundación Cardioinfantil-IC, Bogotá, Colombia.; 4 Endociencia, Bogotá, Colombia.; 5 Yale University, New Haven, United States.

**Keywords:** Diabetes Mellitus Tipo 1, Adolescente, Padres, Relaciones Familiares, Acontecimientos que Cambian la Vida, Enfermería

## Abstract

**Objetivo:**

Explorar las experiencias de adolescentes colombianos y sus padres después del diagnóstico de diabetes mellitus tipo 1 (DM1).

**Método:**

Se realizó un estudio cualitativo descriptivo mediante análisis de contenido en Bogotá, Colombia. Entre mayo y junio de 2024, participaron 36 personas distribuidas en dos grupos focales con 16 adolescentes y tres grupos focales con 23 padres. Las transcripciones de entrevistas semiestructuradas fueron analizadas sistemáticamente para identificar temas comunes entre los participantes. El estudio cumplió con los principios éticos en investigación.

**Resultados:**

Se identificaron seis temas: (1) experiencia de un impacto emocional inicial, (2) cambios en la dinámica familiar, (3) la necesidad de aprender el manejo de la diabetes, (4) dependencia del apoyo externo, (5) importancia de profesionales de salud empáticos y conocedores, y (6) desafíos continuos en el manejo de la condición.

**Conclusión:**

Los hallazgos resaltan que vivir con DM1 requiere adaptación emocional, conductual y social. Las intervenciones integrales centradas en la familia que fomenten la resiliencia, el automanejo y el apoyo emocional son esenciales para mejorar los resultados tanto en los adolescentes como en sus familias.

## INTRODUCCIÓN

La diabetes tipo 1 (DT1) es una enfermedad crónica en la que el páncreas es incapaz de producir insulina. Según la Federación Internacional de Diabetes (FID), en el año 2022 aproximadamente 8,75 millones de personas vivían con esta enfermedad, de las cuales 1,52 millones eran menores de 20 años^(1,2)^. En Colombia, la incidencia de la DT1 es de 6 a 7 por cada 100 000 niños y, concretamente en Bogotá, se diagnostica a aproximadamente 400 niños y/o adolescentes cada año^(3,4)^. Esta afección supone un alto costo para el sistema de salud debido a las complicaciones a largo plazo^(5)^.

El diagnóstico de DT1 durante la adolescencia presenta retos únicos debido a la etapa de desarrollo en el ciclo de vida caracterizada por cambios fisiológicos y psicosociales, que se intensifican por la coexistencia de una enfermedad crónica. Los adolescentes pueden experimentar síntomas de estrés, depresión y ansiedad, lo que afecta directamente al control glucémico y, por lo tanto, repercute negativamente en su calidad de vida^(6,7)^. El manejo de la diabetes requiere ajustes significativos tanto para el adolescente como para su familia, incluyendo actividades como el monitoreo de la glucosa en sangre, la administración de insulina y los ajustes en la dieta y la actividad física. Estos cambios pueden alterar la dinámica entre los adolescentes y sus padres, que son los principales responsables del cuidado diario de sus hijos, lo que contribuye al estrés y la ansiedad debido a la falta de conocimiento sobre la enfermedad, las diferentes prioridades de los adolescentes y los padres, y los cambios que impone en la dinámica familiar^(6,8,9)^. Este proceso genera diversas exigencias tanto para los adolescentes como para los padres, como el apoyo educativo, el apoyo emocional y las necesidades relacionadas con la atención médica.

El manejo de la DT1 es complejo y se requiere una educación continua tanto para los adolescentes como para sus padres sobre el control de la glucosa, la ingesta de carbohidratos y los ajustes constantes de la dosis de insulina en función de la dieta y la actividad física del adolescente. Durante la adolescencia, los cambios hormonales pueden alterar significativamente los niveles de glucosa en sangre, lo que añade otra dificultad al manejo de la enfermedad. Además, el desarrollo de la autonomía durante esta etapa de la vida puede dificultar el cumplimiento de las pautas de tratamiento, especialmente si falta el apoyo adecuado de la familia. Por lo tanto, es esencial que los miembros de la familia también reciban formación para proporcionar el apoyo necesario^(10,11)^.

Un apoyo familiar adecuado para los adolescentes puede conducir a una mejor calidad de vida, un mayor bienestar emocional, un mejor autocuidado y el cumplimiento del tratamiento. Sin embargo, no es fácil para las familias asumir la responsabilidad del cuidado de la DT1, ya que requiere ajustes significativos en el estilo de vida e implica un régimen de cuidados intensivos con el que las familias a menudo no están familiarizadas^(8)^. Un enfoque integral por parte de un equipo interdisciplinario que involucre a los adolescentes y a sus padres puede facilitar la adaptación a la DT1, prevenir complicaciones, construir una nueva normalidad dentro de la familia y crear un entorno familiar saludable^(12,13)^.

En la literatura revisada de los últimos 10 años en Colombia, hay pocas investigaciones destinadas a comprender la experiencia de los adolescentes y sus padres tras un diagnóstico de DT1, especialmente desde una perspectiva cualitativa. Se sabe poco sobre cómo las familias manejan la DT1 a diario y sobre las necesidades no satisfechas que podrían servir de base para intervenciones culturalmente relevantes. Si bien se han realizado numerosas investigaciones a nivel mundial sobre la experiencia de los adolescentes y los padres que viven con DT1, es posible que esos resultados no reflejen plenamente el contexto colombiano. Las diferencias en los sistemas de atención de salud y los valores, creencias y tradiciones culturales pueden influir significativamente en la forma en que se vive y se maneja una enfermedad crónica compleja como la DT1. Por lo tanto, la investigación basada en las realidades locales es fundamental para proporcionar conocimientos y estrategias de apoyo culturalmente relevantes^(9)^. Comprender los factores que influyen en las familias colombianas en el autocontrol de la diabetes puede servir de base para intervenciones culturalmente relevantes e investigaciones futuras. El objetivo de este estudio fue explorar las experiencias de los adolescentes colombianos y sus padres tras un diagnóstico de diabetes tipo 1.

## MÉTODO

### Diseño del Estudio

Se trata de un estudio cualitativo descriptivo guiado por el análisis de contenido^(14)^. Los datos se recolectaron mediante entrevistas a grupos focales. El estudio siguió la lista de verificación de los Criterios Consolidados para Informar sobre Investigaciones Cualitativas (COREQ) a fin de garantizar la transparencia y el rigor en el proceso de presentación de informes^(15)^. El objetivo del estudio fue explorar las experiencias de los adolescentes diagnosticados con diabetes mellitus tipo 1 y sus padres, en particular en relación con los retos que se plantean tras el diagnóstico y durante el control de la enfermedad.

### Lugar

El estudio se llevó a cabo en Bogotá, Colombia, entre mayo y junio de 2024.

Los grupos focales presenciales se llevaron a cabo en las instalaciones de la Asociación Colombiana de Diabetes. Las sesiones virtuales se realizaron a través de la plataforma Microsoft Teams^®^, lo que permitió flexibilidad y accesibilidad para los participantes ubicados en diferentes zonas del país.

### Población y Criterios de Selección

El estudio incluyó a 39 participantes: 16 adolescentes de entre 10 y 17 años con un diagnóstico confirmado de DT1 y 23 padres de adolescentes con DT1. Los criterios de inclusión exigían la capacidad de leer y escribir en español y la disposición a participar en un grupo focal, ya fuera en persona o de forma virtual. Se empleó una estrategia de muestreo por conveniencia y se seleccionó intencionadamente a los participantes para formar grupos focales con homogeneidad en cuanto a edad, sexo y ubicación geográfica, lo que facilitó la expresión de experiencias compartidas.

A pesar de esta agrupación intencionada, la muestra global era heterogénea y abarcaba diversos contextos socioeconómicos y educativos. Para contextualizar, el estudio se centró en adolescentes de diversos entornos familiares y sociales, incluyendo diferentes composiciones familiares, niveles de educación de los padres y entornos residenciales en Bogotá y otras ciudades. Esta diversidad proporciona una perspectiva global de las experiencias y los contextos relevantes para el tema de investigación.

Se invitó por teléfono a participar a cincuenta familias que recibían atención en dos fundaciones para la diabetes. De ellas, 23 aceptaron participar en el estudio. La principal razón para no participar fue la falta de tiempo. La invitación y la explicación de los objetivos del estudio corrieron a cargo del investigador principal, quien garantizó la confidencialidad y la participación voluntaria.

### Recolección de Datos

Para fomentar la expresión abierta de las experiencias, se entrevistó por separado a los adolescentes y a los padres. Las sesiones de los grupos focales siguieron las directrices establecidas e incluyeron los siguientes pasos: a) selección de moderadores con experiencia, b) diseño de una guía de preguntas semiestructuradas y c) organización de cinco grupos focales^(16)^. La guía de preguntas semiestructuradas fue elaborada por un equipo multidisciplinario con experiencia en enfermería, salud pública, psicología clínica y servicios de salud colombianos, con el objetivo de explorar las experiencias y necesidades que surgen con un diagnóstico de DT1. Las sesiones de los grupos focales duraron entre 40 y 90 minutos. Todas las sesiones se grabaron en audio y se transcribieron literalmente, excluyendo los nombres de los participantes para garantizar la confidencialidad, y se asignó un número de código a cada participante. Se tomaron notas de campo sobre aspectos metodológicos y logísticos, y se pidió a los participantes que completaran un breve cuestionario sociodemográfico que incluía preguntas sobre la edad, el sexo, el estatus socioeconómico y la estructura familiar de los participantes, entre otras^(17)^.

### Análisis y Tratamiento de Datos

Para el análisis cualitativo, se utilizó un enfoque de análisis de contenido, que implicó la codificación y categorización sistemática de los datos^(14)^. Para garantizar el rigor metodológico, el investigador principal codificó todas las entrevistas y mantuvo reuniones frecuentes con el equipo multidisciplinario para revisar el proceso de codificación y resolver las discrepancias. A continuación, el equipo organizó los códigos en categorías y subcategorías para identificar los temas clave, utilizando Microsoft Word y Microsoft Excel para la gestión de datos. Se utilizó un enfoque sistemático para garantizar la coherencia en la codificación y la interpretación de todo el conjunto de datos.

La credibilidad se garantizó mediante el uso de códigos in vivo, el análisis colaborativo y la validación de los resultados con los participantes utilizando la técnica de “verificación por los miembros”. Además, se alcanzó la saturación teórica, lo que garantizó que no surgieran nuevos temas o ideas a partir de los datos, lo que reforzó la profundidad y la exhaustividad del análisis. En este enfoque, los resultados consolidados se devolvieron los participantes para que verificaran su exactitud. Además, se aplicaron estrategias de triangulación metodológica y teórica para mejorar la transferibilidad de los resultados^(18)^. Este proceso dio lugar a expresiones como las siguientes:

Participante 1: *Excelente, sin duda. Ha logrado resumir todo maravillosamente y, de hecho, como siempre he dicho y sugerido a la asociación, para empoderar a estos niños y hacerlos productivos en el futuro, necesitamos fortalecer la columna vertebral de toda esta situación, que son las madres. Muchas gracias, ha sido realmente muy conmovedor*.

Participante 2: *Una síntesis perfecta de todo lo que ocurre en el proceso tras el diagnóstico y la toma de conciencia de que tienen que vivir con una enfermedad crónica de por vida. Creo que está muy bien hecho*.

### Aspectos Éticos

El estudio se ajustó a las normas éticas descritas en la Resolución 008430 y recibió la aprobación ética con el número de registro 003 el 19 de febrero de 2024 del Comité de Ética de la Escuela de Enfermería y Rehabilitación, así como la Acta n.° 202 el 22 de mayo de 2024 de la Facultad de Ciencias del Comportamiento de la Universidad de La Sabana. Se obtuvo el consentimiento informado por escrito de los padres y el consentimiento informado por escrito de los adolescentes. Se empleó un sistema de codificación alfanumérica para garantizar el anonimato de los participantes.

## RESULTADOS

Participaron en el estudio dieciséis adolescentes con diabetes tipo 1, con una edad media de 13,6 años (DE = 2,4). Entre ellos, el 68,8% (n = 11) eran mujeres. La mayoría de los adolescentes cursaban el décimo grado, lo que representaba el 31,3% (n = 5) de la muestra; en cuanto al estatus socioeconómico, el 43,8% (n = 7) pertenecía a un estrato socioeconómico bajo según la clasificación colombiana. Además, la mitad de los participantes (50,0%, n = 8) declararon vivir con un solo progenitor. La mayoría (81.3%, n = 13) residía en Bogotá (Tabla 1).

**Tabla 1:** Características sociodemográficas de los adolescentes – Colombia, 2024.

Variable	Categorías	N (16)	%
Variables categóricas			
Sexo	Hombre	5	31,3
	Mujer	11	68,8
Nivel educativo	Quinto	2	12,5
	Sexto	2	12,5
	Séptimo	1	6,3
	Octavo	1	6,3
	Noveno	3	18,8
	Décimo	5	31,3
	Undécimo	2	12,5
Estatus socioeconómico	Bajo	7	43,8
	Medio-bajo	5	31,3
	Medio-alto	3	18,8
	Alto	1	6,3
Estructura familiar	Madre y padre	6	37,5
	Solo madre o solo padre	8	50,0
	Otras personas	2	12,6
Lugar de residencia	Bogotá	13	81,3
	Otras ciudades	3	18,9
Variables continuas			
	Media (DE)	Min–Max	
Edad (años)	13,6 (2,4)	10–18	

Fuente: Elaboración propia.

Además, veintitrés padres de adolescentes con DT1 también participaron en el estudio, de los cuales el 82.6% (n = 19) eran mujeres. En cuanto al nivel educativo, el 30.4% (n = 7) había completado la educación técnica, seguido del 21.7% (n = 5) con educación secundaria y el 21.7% (n = 5) con educación universitaria. En cuanto al estatus socioeconómico, la mayoría de los padres pertenecían a un estrato socioeconómico bajo (34.8%, n = 8), según la clasificación colombiana. El estado civil se distribuía de la siguiente manera: el 34.8% estaba casado, el 26.1% era soltero y el 13.0% estaba divorciado, en unión libre o separado. En cuanto a la composición familiar, el 65.2% informó que vivía con ambos padres e hijos, el 21.7% con un padre e hijos y el 13.0% con abuelos u otros miembros del hogar. La mayoría (73.9%) residía en Bogotá, mientras que el 26.1% vivía en otras ciudades (Tabla 2).

**Tabla 2:** Características sociodemográficas de los padres – Colombia, 2024.

Variable	Categorías	N (23)	%
Variables categóricas			
Sexo	Hombre	4	17,4
	Mujer	19	82,6
Nivel Educativo	Secundaria incompleta	4	17,4
	Secundaria completa	5	21,7
	Educación técnica	7	30,4
	Universidad	5	21,7
	Post graduación	2	8,7
Estatus socioeconómico	Baja-baja	1	4,3
	Baja	8	34,8
	Media baja	7	30,4
	Media alta	3	13,1
	Alta	3	13,1
	Elevada	1	4,3
Estado civil	Soltero	6	26,1
	Casado	8	34,8
	Divorciado	3	13,1
	Unión civil	3	13,1
	Separados	3	13,1
Estructura familiar	Madre, padre e hijos	15	65,2
	Solo la madre o el padre y los hijos.	5	21,7
	Mamá, papá, hijos y abuelos	1	4,3
	Otras personas	2	8,7
Ocupación	Estudiante	2	8,7
	Empleado	9	39,1
	Desempleado	7	30,4
	Dueña de casa	3	13,1
	Autónoma	2	8,7
Lugar de residencia	Bogotá	17	73,9
	Otras ciudades	6	26,1
Variables continuas			
	Media (DE)	Min–Max	
Edad (años)	45,3 (9,5)	30–68	

Fuente: Elaboración propia.

### Análisis: Temas

Los participantes informaron que vivir con diabetes tipo 1 era un proceso complejo caracterizado por múltiples retos y necesidades. Este proceso, ilustrado en la Figura 1, comienza con un impacto emocional significativo tanto en los adolescentes como en sus padres. A esto le siguen cambios en la dinámica familiar y la necesidad de desarrollar habilidades para manejar la enfermedad. Estos temas están interconectados y forman un marco conceptual que refleja las experiencias vividas por los participantes. En el análisis de los datos, se identificaron temas comunes, más que diferencias, tanto entre los adolescentes como entre los padres. Esta representación destaca las etapas y los retos clave, lo que permite una comprensión holística de las experiencias de los participantes.

**Figura 1:** Vivir con diabetes tipo 1: perspectiva de los adolescentes y los padres.

Fuente: elaboración propia.

### Tema 1: La Experiencia del Impacto Inicial Emocional

Los adolescentes y los padres que participaron en este estudio informaron que el diagnóstico de DT1 tuvo un gran impacto en sus vidas.

Los padres de los adolescentes a menudo experimentaron emociones complejas en el momento del diagnóstico, incluyendo miedo y confusión debido a su limitada comprensión de cómo manejar esta compleja condición de salud. Con frecuencia expresaron una preocupación constante por las posibles complicaciones y también se sintieron culpables por no poder evitar los retos a los que se enfrentaban sus hijos como resultado del diagnóstico. Estos sentimientos de miedo, duda y culpa se intensificaron por la falta de conocimiento y las situaciones traumáticas que vivieron tanto los padres como sus hijos en los hospitales o durante el diagnóstico y tratamiento iniciales.

A continuación se muestran algunos ejemplos de extractos de padres:

Papá 1: *J comenzó a los 10 años, mmm, siempre fue un impacto para nosotros porque estuvo en la UCI. Así que estuvo en la UCI. Entonces, se complicó un poco, así que fue un poco complejo desde ese lado*.

Mamá 4: *Entonces rompí a llorar. Realmente rompí a llorar. Dije: “Dios mío, ¿pero por qué?”. Dije: “Lo hice todo”. Me culpé a mí misma*.

Mamá 7: *Es difícil: con acoso, sin acoso, con amor, con deportes, sin deportes, en cualquier circunstancia, esto es muy difícil. Con dinero, sin dinero, quiero decir, es difícil desde cualquier punto de vista*.

Los adolescentes a menudo experimentaban sentimientos de confusión y luchaban por adaptarse a la vida con DT1. Al principio, informaban de sentimientos de resistencia y negación hacia el diagnóstico. Afrontar los cambios en el estilo de vida era especialmente difícil, sobre todo debido a las restricciones alimentarias, el uso regular de insulina y glucómetros, y la necesidad de un autocontrol continuo. Además, sentían una mayor responsabilidad y podían sentirse abrumados por las exigencias del monitoreo de su enfermedad. Esta situación se complicaba aún más por las interacciones con sus amigos y compañeros, ya que algunos adolescentes sufrían acoso escolar. Estas experiencias sociales negativas añadían una carga emocional, lo que les dificultaba aún más adaptarse a su nueva realidad.

Adolescente 2: *Bueno, para mí fue un poco difícil acostumbrarme porque a veces quiero comer dulces o algo así y no puedo. No me inyectaba la insulina ni me medía los niveles de glucosa, pero aprendí con mi mamá porque veía que se cansaba de luchar por mí*.

Adolescente 4: *Tengo 15 años. Bueno, para mí fue difícil, pero ahora en la escuela algunos compañeros me ayudan. Muchos de ellos también me recuerdan que lleve todo lo que necesito, así que me ayudan un poco*.

Adolescente 5: *Bueno, a veces sientes presión porque si cometes un error, te sientes mal, ya que un error puede provocar hipoglucemia, así que es difícil*.

Adolescente 8: *Fue un poco difícil, pero pensé que no tenía ningún problema. Sin embargo, siempre es difícil con los compañeros de clase. A veces quieren intimidarte y esas palabras duelen*.

### Tema 2: Sentir el Cambio en la Dinámica Familiar

El diagnóstico de DT1 contribuyó a cambios significativos en la dinámica familiar, particularmente en relación con los ajustes en el estilo de vida, con especial énfasis en la dieta y los hábitos de sueño y descanso.

Los padres informaron haber experimentado cambios en sus rutinas diarias, y la preocupación constante por el manejo adecuado de la enfermedad se convirtió en una responsabilidad las 24 horas del día. Esta nueva dinámica a menudo afectaba a su trabajo, descanso y vida social. Además, podían surgir conflictos familiares debido a los retos asociados a la adolescencia, con padres que se percibían a sí mismos como demasiado estrictos o sobreprotectores, lo que creaba tensión en su relación con sus hijos. A pesar de estas dificultades, los padres expresaron un fuerte compromiso con el cuidado y la protección de sus hijos. Se adaptaron a estos cambios y buscaron activamente soluciones para mejorar el bienestar de su familia.

A continuación se muestran algunos ejemplos de códigos de padres:

Papá 2: *Ha causado algunos conflictos porque acabo siendo el papá estricto, el papá enojado. “¿Por qué no te has puesto la insulina? ¿Qué pasa? ¿Quieres ir al hospital?*

Mamá 4: *En casa, si hay alimentos prohibidos, es porque no están en la casa, así de simple. El resto de los alimentos de la casa, todos podemos comerlos*.

Mamá 5: *Por lo general, en esos días, debido a la actividad física, por la noche, cuando están descansando, sus niveles tienden a bajar, bajar y bajar. Así que se despiertan 1, 2, 3, 4 veces, y ya se sabe que el sueño interrumpido es agotador*.

Los adolescentes informan que, tras ser diagnosticados con DT1, sus padres les insisten en que deben asumir la responsabilidad de su propio cuidado. Por ejemplo, deben monitorear sus niveles de glucosa con regularidad, lo que a menudo interrumpe sus actividades diarias en el ámbito social y escolar. Por lo tanto, los adolescentes se adaptan constantemente a nuevos retos, buscando el equilibrio en su vida cotidiana.

Adolescente 3: *En la escuela, mis padres siempre me piden mis lecturas de glucosa, me dicen que les envíe mis lecturas de glucosa y, a veces, resulta molesto*.

Adolescente 4: *Creo que también es un poco molesto para mí porque siempre están ahí, controlando, vigilando, comprobando si me he tomado la lectura de glucosa, si me he inyectado la insulina*.

### Tema 3: Necesidad de Aprender a Controlar la Condición de Salud

Los adolescentes y los padres percibían el control de la DT1 como un proceso complejo que requería una educación integral por parte de los profesionales sanitarios. Consideraban que la educación era esencial para ayudarles a adaptarse a los cambios en el estilo de vida asociados a la DT1. Tanto los adolescentes como los padres expresaron la necesidad de aprender a dosificar correctamente las unidades de insulina, contar los carbohidratos y controlar eficazmente las crisis de hiperglucemia e hipoglucemia.

Los padres, en particular, informaron de que necesitaban una educación completa y continua desde el momento del diagnóstico. Muchos señalaron la falta de orientación clara y detallada sobre el control de la enfermedad de sus hijos, especialmente en áreas críticas como el recuento de carbohidratos, el control de la dieta, la administración de insulina y el uso de tecnologías como las bombas de insulina y los glucómetros. Además, los padres tuvieron que desarrollar estrategias para una crianza positiva, fomentando la autonomía y evitando enfoques basados en el miedo. Para llenar estos vacíos, los padres a menudo buscaban información a través de las redes sociales y los grupos de apoyo para contrarrestar el estigma y la desinformación en su entorno. Los padres enfatizaron la importancia de contar con programas educativos estructurados y accesibles que abordaran tanto los aspectos médicos como psicosociales del manejo de la diabetes en los adolescentes, ya que consideraban que estos programas les habrían ayudado a afrontar los retos de la DT1 con más confianza y competencia.

A continuación se presentan algunos ejemplos de extractos de adolescentes y padres:

Mamá 4: *No hay mucha formación sobre el recuento de carbohidratos, eh, cómo manejarlo*.

Mamá 7: *Cuando diagnostican al niño, nadie te dice qué hacer. Solo te dicen: “Comprueba sus niveles de glucosa. Si están dentro de este rango, entonces ya está”, pero nadie te educa realmente como padre*.

Mamá 6: *Bueno, no sé, creo que lo ideal sería que esto no se convirtiera en una rutina más, sino que la educación que se imparte a los padres de niños con esta afección fuera realmente atractiva, de una manera lúdica, para que ellos también se involucren y aprendan*.

Los adolescentes expresaron la necesidad de recibir orientación continua y completa sobre su afección, incluso años después del diagnóstico inicial, y destacaron que esta debería ser accesible, amigable y detallada, e incluir información sobre el tratamiento, consejos prácticos y soluciones para el manejo diario. Destacaron la importancia de una educación nutricional adecuada, en particular centrada en el recuento de carbohidratos. Además, subrayaron el requisito esencial de la psicoeducación dentro de su entorno social, con el objetivo de concienciar sobre los riesgos y el manejo de situaciones como la hipoglucemia y la hiperglucemia, para garantizar un entorno seguro y de apoyo.

Adolescente 3: *“A veces necesitamos orientación porque, aunque llevamos muchos años con diabetes, todavía no sabemos todo lo que requiere”*.

Adolescente 4: *Una introducción más orientada a la familia, porque en el grupo, a veces las mamás que tienen hijos recién diagnosticados solo reciben el diagnóstico, pero no obtienen una introducción completa sobre lo que realmente es la diabetes, algo cómodo, con consejos, soluciones y todo eso*.

Adolescente 5: *Creo que lo que realmente falta es educación sobre la alimentación, porque a veces lo restringen todo y básicamente no puedes comer nada. Creo que deberían centrarse más en enseñar a contar los carbohidratos para evitar restringir completamente la comida*.

### Tema 4: Necesidad de Apoyo Social Para Afrontar la Vida con DT1

Los adolescentes y los padres informaron de que el apoyo social era importante para la adaptación a la DT1. Los padres de los adolescentes expresaron la necesidad de apoyo emocional para afrontar el estrés y la ansiedad constantes asociados al cuidado de sus hijos. Destacaron la importancia de conectar con otros padres en situaciones similares para compartir experiencias y sentirse más seguros. También necesitaban apoyo para el manejo diario de la diabetes, incluyendo tareas como el monitoreo nocturno, la administración de medicamentos y el recuento de carbohidratos. Los padres destacaron que el apoyo familiar y social era fundamental para mantener su propio bienestar mental. Muchos padres también informaron de que encontraban fuerza en sus creencias espirituales, que les proporcionaban consuelo y orientación durante todo el proceso de adaptación. A continuación se muestran algunos ejemplos:

Mamá 5: *Especialmente porque mi papá casi fallece hace seis meses y era prácticamente tanto un padre como un abuelo para mi hija. Por lo tanto, mi papá también desempeñó un papel importante; fue un apoyo fundamental para nosotros porque siempre se aseguraba de que todo estuviera en orden con los trámites legales*.

Mamá 6: *Conocimos a más mamás, y esa orientación fue vital, realmente vital, porque ahora mi hija tiene algunas amigas con la misma afección. Así que, los fines de semana, las invitaba a casa, hablaba con sus mamás y nos reuníamos*.

Mamá 7: *Te rindes a Dios, sí, te lo digo, nos rendimos, como dicen, a Dios, pidiéndole que nos ayude, que me dé fuerzas, que me dé paz para poder tomar decisiones acertadas cuando las necesito*.

Los adolescentes informaron haber experimentado una presión social que les provocó angustia emocional, lo que fomentó la necesidad de apoyo social que les hiciera sentir cómodos al compartir sus experiencias con compañeros que se enfrentaban a situaciones similares. Se esforzaban por ser aceptados, sentirse comprendidos y mantener una sensación de normalidad en su entorno social. Sin embargo, se dieron cuenta de que a menudo es difícil para los demás comprender plenamente la complejidad de vivir con DT1. Aunque los adolescentes reconocían que adaptarse al diagnóstico de diabetes era un reto, destacaban el papel crucial del apoyo de sus padres para ayudarles a adaptarse a la nueva realidad de vivir con ella.

A continuación se muestran ejemplos de extractos de adolescentes:

Adolescente 3: *En la escuela nunca tuve amigos porque siempre me decían que era contagioso, aunque nunca lo es, o me llamaban “la chica rara”*.

Adolescente 6: *Más bien ponerse en nuestra situación como adolescentes, porque queremos vivir lo más normalmente posible y, a veces, nos descuidamos. Olvidamos que tenemos una enfermedad y que tenemos que ser responsables, algo así*.

Adolescente 3: *La diabetes no es una enfermedad, es una condición que Dios envía con un propósito en la vida, y siento que aceptarlo tampoco fue tan fácil*.

### Tema 5: Necesidad de Profesionales de la Salud Empáticos y con Conocimientos

Los padres y los adolescentes informaron de experiencias predominantemente negativas con los profesionales de la salud que participaron en su tratamiento inicial.

Los padres expresaron que los profesionales de la salud empáticos y conocedores del manejo de la DT1 podrían haber desempeñado un papel clave para ayudarles a comprender y adaptarse a los retos de salud asociados a la afección. Destacaron la falta de apoyo profesional adecuado y señalaron que muchos profesionales de la salud no proporcionaban una orientación clara y completa sobre el manejo de la afección. También observaron que algunos profesionales carecían de la empatía y la sensibilidad necesarias para atenderlos a ellos y a sus hijos, y describieron ciertas interacciones como “abruptas y poco comprensivas”. Como resultado, muchos padres recurrieron a profesionales privados o fundaciones para obtener la orientación y el apoyo que a menudo no encontraban en el sistema de atención de salud público.

Aquí presentamos algunos ejemplos:

Mamá 5: *¿Eh? La llevé a la clínica y el médico que nos atendió fue extremadamente grosero. Me dijo directamente a la cara que mi hija podía morir, y fue un shock. Creo que todas las mamás, cuando nos dicen “tu hija tiene esto y podría morir”, sentimos que es un golpe muy duro*.

Papá 3*: Cuando tuvimos que hacernos un análisis de sangre, el primero se hizo a través del seguro médico. La técnica de laboratorio también fue muy grosera, le gritó al niño porque lo vio llorar. Creo que en ese aspecto debería haber un poco más de sensibilidad con los niños*.

Mamá 5: *Siento que, sí, lo verdaderamente esencial es encontrar profesionales que tengan ética, que realmente amen su profesión y que se involucren en el proceso de una enfermedad que hoy en día es tan común*.

Mamá 4: *El psicólogo al que acudimos, solo una cita con el psicólogo, y no me gustó. No me gustó el trato ni la forma en que nos trataron… Le dije que no, que mi bebé es solo un bebé pequeño… La nutricionista de la compañía de seguros médicos, tampoco… Nunca volví a ir… Esa mujer iba a matar de hambre a mi hija; básicamente, no podía comer nada, solo agua*.

Los adolescentes también informaron que necesitaban comprensión, apoyo emocional y una comunicación más empática por parte de los profesionales de la salud. Prefieren profesionales que les proporcionen orientación y apoyo continuos para gestionar los retos emocionales y sociales de vivir con diabetes.

Aquí presentamos un ejemplo:

Adolescente 1: *Creo que debería haber apoyo para comprender que, aparte de eso, digamos, cuando no conocemos a nadie que nos haya orientado adecuadamente sobre la aplicación de insulina, cómo controlar la enfermedad, la dieta y el recuento de carbohidratos*.

### Tema 6: Vivir con los Retos Constantes en el Manejo de la DT1

El manejo de la DT1 planteó retos importantes para los adolescentes y sus padres debido a las constantes fluctuaciones de los niveles de azúcar en sangre, el control de la insulina y el recuento de carbohidratos necesario para el control de la dieta.

Los padres de adolescentes con diabetes se enfrentaban a retos adicionales a la hora de controlar la enfermedad en diversos entornos sociales. A menudo debían depender en gran medida de prácticas de monitoreo como la monitorización continua de la glucosa, las pruebas de glucosa en sangre y otras para mantener los niveles de glucosa en sangre de sus hijos dentro de los límites deseados. Los eventos sociales fuera de casa eran especialmente difíciles, ya que a los padres les resultaba complicado calcular el contenido de carbohidratos de los alimentos que no habían preparado ellos mismos, lo que les provocaba ansiedad y les obligaba a elaborar planes de contingencia. Además, la ansiedad de los padres aumentaba cuando los adolescentes participaban en actividades independientes, como reuniones sociales, ya que esto requería confiar en la capacidad de sus hijos para controlar la enfermedad de forma eficaz. A continuación se muestran algunos ejemplos:

Mamá 4: *Sin embargo, en su lonchera lleva insulina. En su lonchera y en sus cosas lleva el medidor de glucosa, agujas, tomamos todas estas precauciones. Si necesita inyectarse, ella ya sabe cómo hacerlo*.

Mamá 2: *Porque llega un momento en que llega la adolescencia y empiezan las salidas con los amigos. Ese fue probablemente el momento más caótico que he vivido. Este dilema era tan abrumador que le dije: “No, llévate lo que quieras”. Él es responsable de cuidarse a sí mismo; sabe perfectamente cómo manejarse*.

Los adolescentes se enfrentaban a numerosos retos a la hora de controlar la diabetes, como inyectarse insulina y medir sus niveles de glucosa en la escuela, a menudo en entornos en los que carecían del apoyo adecuado. También se encontraban con dificultades diarias relacionadas con el consumo de alimentos, ya que la mayoría de las personas no están familiarizadas con el recuento de carbohidratos. Los adolescentes afirmaban tener que asumir la responsabilidad de equilibrar su deseo de disfrutar de los alimentos que les gustan con la necesidad de mantener el control del azúcar en sangre. Esto podía dar lugar a decisiones difíciles y a sentimientos de aislamiento.

Adolescente 2: *Bueno, para mí fue un poco difícil acostumbrarme porque a veces quiero comer dulces o algo así y no puedo. No me ponía la insulina ni me medía los niveles de glucosa, pero aprendí con mi mamá porque veía que se cansaba de luchar por mí*.

Adolescente 5: *Es muy fácil equivocarse al contar los carbohidratos, y más aún con lo que te estoy diciendo. Si pudieras servir una porción perfectamente medida de arroz, carne y todo lo demás, exactamente 35 gramos, sería perfecto*.

## DISCUSIÓN

En esta investigación, se identificaron seis temas centrales relacionados con las experiencias y necesidades de los adolescentes y padres colombianos que viven con DT1. El primer tema destaca que, en el momento del diagnóstico, tanto los padres como los adolescentes se enfrentaron a una situación difícil debido a una enfermedad crónica compleja, como la falta de conocimiento sobre la enfermedad y su manejo, lo que creó desafíos físicos y emocionales. Estos hallazgos coinciden con un estudio realizado en Brasil, que informó que cuando los adolescentes son diagnosticados con diabetes, se someten a un proceso complejo que implica hospitalizaciones, pruebas médicas y el profundo impacto que el diagnóstico tiene en sus vidas^(18)^.

De manera similar, en una investigación realizada en Brasil, el diagnóstico de DT1 provocó sentimientos de preocupación, tristeza e ira entre los participantes debido a la naturaleza inesperada de la situación y su conocimiento limitado sobre la enfermedad, lo que condujo a momentos de angustia emocional^(19)^. El manejo de la DT1 plantea desafíos significativos para las familias, ya que el impacto emocional, la falta de experiencia y la incertidumbre de los padres en el manejo de la afección pueden intensificar el dolor asociado con la aceptación del diagnóstico. Esto a menudo interfiere en la vida cotidiana y puede requerir apoyo especializado^(20)^.

El segundo tema identificado en este estudio se centró en los cambios experimentados por los adolescentes y los padres durante el proceso de diagnóstico. Estos cambios incluían ajustes en la dieta, la actividad física, la medicación y los hábitos de sueño. Investigaciones anteriores han demostrado que el diagnóstico de DT1 altera significativamente la vida de los adolescentes y sus familias. Los adolescentes deben adoptar nuevos hábitos, al tiempo que a menudo se enfrentan a retos para adherirse al tratamiento. Las restricciones dietéticas, el monitoreo constante de la glucosa y los chequeos regulares pueden dar lugar a dificultades en las responsabilidades de autocuidado^(21)^.

El tercer tema destacado por los participantes fue la necesidad continua de educación sobre temas como el recuento de carbohidratos, el control de la dieta, la administración de insulina y el uso de tecnologías como las bombas de insulina y el monitoreo continuo de la glucosa. Educar a las familias sobre cómo cuidar a los adolescentes con una enfermedad crónica no solo alivia la carga del cuidado, sino que también mejora su calidad de vida^(19)^. Sin embargo, la educación no es algo puntual; a medida que los niños y adolescentes crecen, sus necesidades médicas y de desarrollo evolucionan, lo que requiere un acceso continuo a recursos educativos fiables. Las investigaciones subrayan la importancia de los programas de educación continua sobre la diabetes que proporcionan información actualizada y apoyo adaptado a las diferentes etapas de la vida^(20)^. Proporcionar información clara y personalizada a los padres y adolescentes sobre cómo manejar estos cambios puede ayudarles a comprender mejor su situación, reducir la desregulación emocional y promover el cumplimiento del tratamiento, lo que en última instancia mejora el control metabólico^(8,11,21)^.

El cuarto tema identificó el apoyo emocional que necesitan los adolescentes y los padres. Ambos grupos expresaron la importancia de recibir apoyo de los familiares y otras personas que se enfrentan a retos similares para adaptarse mejor a la vida con diabetes. Del mismo modo, un estudio realizado en China reveló que los participantes valoraban el apoyo emocional de familiares y compañeros con experiencias similares, ya que les brindaba la oportunidad de debatir y aprender estrategias para controlar la diabetes. Es esencial abordar las preocupaciones y demandas de los adolescentes y sus cuidadores, y crear espacios para el debate abierto^(22,23,24)^. El apoyo social se ha reconocido como una herramienta fundamental para mejorar el bienestar emocional de los adolescentes con DT1. Un fuerte apoyo familiar influye positivamente en la autoeficacia y el bienestar emocional, mitigando el estrés de vivir con la enfermedad^(23)^. Esta necesidad de apoyo se extiende más allá del diagnóstico inicial a todo el proceso de control. Las organizaciones profesionales, los grupos de apoyo social locales y las redes en línea desempeñan un papel fundamental a la hora de conectar a los padres y adolescentes con DT1, ofreciéndoles apoyo emocional y educativo. Además de las plataformas profesionales, como la Asociación Americana de Diabetes, los grupos de apoyo comunitarios proporcionan orientación culturalmente relevante y un sentido de pertenencia. Los grupos de redes sociales también facilitan el intercambio de experiencias, lo que promueve la adaptación y mejora la adherencia al tratamiento^(25,26)^.

El quinto tema destaca la importancia de contar con profesionales de la salud competentes y empáticos para ayudar a las familias a adaptarse a la DT1. Este hallazgo concuerda con investigaciones anteriores, en las que los adolescentes informaron de un apoyo insuficiente en el momento del diagnóstico y de sentimientos de agobio por la responsabilidad^(27)^. Los profesionales sanitarios especializados deben ser capaces de explicar la interacción entre la glucosa, la dieta y la insulina, al tiempo que proporcionan recomendaciones nutricionales específicas^(10,25,28)^. En un estudio, los adolescentes con DT1 describieron experiencias negativas, como interacciones despersonalizadas y una comunicación centrada principalmente en sus padres. También hicieron hincapié en la necesidad de disponer de más tiempo para discutir sus preocupaciones con los profesionales sanitarios^(27)^.

Por último, el sexto tema abordó los retos a los que se enfrentan los padres y los adolescentes, incluido el estrés relacionado con el control de la dieta y los niveles de glucosa en sangre, especialmente durante los eventos sociales en los que es difícil contar los carbohidratos. Investigaciones anteriores señalaron que a los participantes les costaba seguir su dieta en entornos con dulces y bebidas azucaradas, ya que este tipo de alimentos resultan especialmente tentadores para los adolescentes^(28)^. Recientemente, los avances en el recuento de carbohidratos han permitido a los adolescentes comer una mayor variedad de alimentos^(27)^. Sin embargo, al igual que ocurre con los adolescentes sin diabetes, esto puede conducir a un consumo excesivo de calorías y al aumento de peso. Por lo tanto, es esencial comprender los retos a los que se enfrentan los adolescentes y los padres en relación con las restricciones dietéticas y las inyecciones de insulina, ya que pueden afectar a la autoimagen, la adaptación y las actividades diarias de los adolescentes. Es fundamental proporcionar estrategias de afrontamiento eficaces para controlar la DT1^(28)^.

Los resultados de este estudio proporcionan información valiosa para que los profesionales de la salud diseñen y apliquen intervenciones adaptadas a las exigencias específicas de la población sudamericana. Es esencial un enfoque integral y multidisciplinario, con especial énfasis en las intervenciones de apoyo en el momento del diagnóstico y la educación continua. Además, ofrecer recursos comunitarios y en línea para que los padres reciban apoyo emocional y social puede ayudar tanto a los adolescentes recién diagnosticados con DT1 como a sus familias. También se considera esencial prestar mayor atención a la detección rutinaria del malestar psicológico, los síntomas depresivos y la ansiedad en la atención clínica, según las directrices de atención establecidas^(29)^. Abordar estas demandas mediante la educación continua, el apoyo psicosocial y la atención profesional especializada será crucial para satisfacer las necesidades holísticas de esta población en los países sudamericanos.

Al centrarse en los adolescentes colombianos y sus familias, este estudio aporta una perspectiva culturalmente específica que añade profundidad a la comprensión global de la DT1. Las familias colombianas tienden a involucrarse mucho en el manejo diario de las enfermedades crónicas, y ambos padres desempeñan un papel activo en el apoyo a los adolescentes. Esto subraya la necesidad de intervenciones educativas que incluyan a toda la unidad familiar, en lugar de centrarse únicamente en el adolescente. Además, los adolescentes colombianos pueden beneficiarse de evaluaciones psicosociales rutinarias en entornos clínicos. Si bien las normas internacionales de atención recomiendan la detección de síntomas depresivos, angustia relacionada con la diabetes y ansiedad, en Colombia no se aplica de manera sistemática, y las vías de derivación a servicios de apoyo para los adolescentes afectados siguen siendo limitadas. Estos hallazgos ponen de relieve la importancia de fortalecer el sistema de atención de salud colombiano mediante la integración de la educación centrada en la familia, la evaluación psicosocial y el acceso a un apoyo adecuado en materia de salud mental en la atención rutinaria de los adolescentes con DT1.

Las intervenciones anteriores han tenido como objetivo promover conductas de autocuidado y control metabólico en los adolescentes^(5,30)^, así como estrategias centradas en la familia diseñadas para mejorar la calidad de vida tanto de los adolescentes como de sus padres, mejorar la comunicación familiar y fomentar las habilidades para resolver problemas^(27,28,29)^. Estos hallazgos subrayan la necesidad de seguir desarrollando y aplicando enfoques holísticos que integren la gestión médica, la atención preventiva, la educación y el apoyo emocional en los sistemas de salud de todo el mundo. En algunos países, las enfermeras pueden obtener la certificación como educadoras en diabetes, lo que les permite brindar el apoyo integral que necesitan los adolescentes con DT1 y sus familias, mientras que en otros, los nutricionistas desempeñan un papel clave en la enseñanza de habilidades como el recuento de carbohidratos. Explorar la disponibilidad e integración de estos recursos dentro del sistema de salud colombiano podría mejorar la calidad de la atención y los resultados para esta población. En el futuro se deberían realizar investigaciones sobre la implementación de una atención integral y multidisciplinaria en diferentes sistemas de salud, y se deberían modificar las estrategias basadas en la evidencia y centradas en la familia en diferentes contextos culturales.

Por último, es importante tener en cuenta las limitaciones metodológicas de este estudio a la hora de interpretar los resultados. La dinámica social de los grupos focales puede haber dado lugar a un sesgo de deseabilidad social, en el que los participantes ajustaron sus respuestas para ajustarse a las normas del grupo o a las expectativas percibidas del moderador. Además, el pequeño tamaño de la muestra de una sola zona geográfica limita la generalización; sin embargo, los resultados son coherentes con la bibliografía existente y pueden orientar el desarrollo de intervenciones culturalmente relevantes.

## CONCLUSIÓN

La experiencia de vivir con DT1 está marcada por importantes ajustes emocionales y de comportamiento tanto para los adolescentes como para sus padres. Para los adolescentes, el diagnóstico altera su sentido de la normalidad, lo que les lleva a sentir incertidumbre, frustración y resistencia al tratamiento. Se enfrentan a retos para mantener las interacciones sociales, cumplir las restricciones dietéticas e integrar el monitoreo de la glucosa en su vida cotidiana. Los padres, por su parte, experimentan una mayor ansiedad, miedo a las complicaciones y una abrumadora sensación de responsabilidad por el bienestar de sus hijos adolescentes. La necesidad constante de vigilancia, junto con la preocupación por la independencia y el futuro de sus hijos, a menudo conduce a un malestar emocional. La falta de una educación integral basada en la familia sobre el manejo de la DT1 agrava aún más estos retos, lo que convierte el cuidado diario en una fuente de tensión dentro de la familia. Las intervenciones que abordan tanto la adaptación emocional como las habilidades de autocontrol son cruciales para promover una experiencia más positiva de vivir con DT1, fomentar la resiliencia y mejorar la calidad de vida tanto de los adolescentes como de sus padres.

## Data Availability

El conjunto de datos que respaldan las conclusiones de este estudio está disponible previa solicitud al autor correspondiente.
